# High frequency of SPG4 in Taiwanese families with autosomal dominant hereditary spastic paraplegia

**DOI:** 10.1186/s12883-014-0216-x

**Published:** 2014-11-25

**Authors:** Min-Yu Lan, Yung-Yee Chang, Tu-Hseuh Yeh, Szu-Chia Lai, Chia-Wei Liou, Hung-Chou Kuo, Yih-Ru Wu, Rong-Kuo Lyu, Jen-Wen Hung, Ying-Chao Chang, Chin-Song Lu

**Affiliations:** Center for Parkinson’s Disease, Kaohsiung Chang Gung Memorial Hospital, Kaohsiung, Taiwan; Department of Neurology, Kaohsiung Chang Gung Memorial Hospital and Chang Gung University College of Medicine, Kaohsiung, Taiwan; Neuroscience Research Center, Chang Gung Memorial Hospital, Linkou, Taoyuan Taiwan; Section of Movement Disorder, Department of Neurology, Chang Gung Memorial Hospital, Linko Medical Center and Chang Gung University College of Medicine, 5 Fu-Shin St, Kwei-Shan, Taoyuan 333 Taiwan; Healthy Aging Research Center, Chang Gung University, Taoyuan, Taiwan; Department of Neurology, Chang Gung Memorial Hospital Linko Medical Center and Chang Gung University College of Medicine, Taoyuan, Taiwan; Department of Rehabilitation, Kaohsiung Chang Gung Memorial Hospital and Chang Gung University College of Medicine, Kaohsiung, Taiwan; Department of Pediatrics, Kaohsiung Chang Gung Memorial Hospital and Chang Gung University College of Medicine, Kaohsiung, Taiwan; School of Traditional Chinese Medicine, College of Medicine, Chang Gung University, Taoyuan, Taiwan

**Keywords:** Hereditary spastic paraplegia, SPG4, *SPAST*, Spastin, AAA cassette

## Abstract

**Background:**

Hereditary spastic paraplegias (HSPs) are a group of neurodegenerative diseases characterized by progressive spasticity and weakness of the lower limbs. SPG4, SPG3A and SPG31 are the three leading causes of autosomal dominant (AD) HSPs.

**Methods:**

A total of 20 unrelated AD-HSP families were recruited for clinical and genetic assessment. Detection of mutations in SPG4, SPG3A and SPG31 genes was conducted according to a standard protocol. Genotype-phenotype correlations and determinants for disease severity and progression were analyzed.

**Results:**

Mutations in the SPG4 gene (*SPAST*) were detected in 18 (90%) of the AD-HSP families. Mutations in SPG4, SPG3A and SPG31 genes were not detected in the remaining two families. Considerable variations in clinical features were noted, even for mutation carriers from the same family. Mutations causing complete loss of the spastin AAA cassette were associated with earlier onset of disease (20 ± 18 years) compared with those with preservation of partial or total AAA cassette (32 ± 19 years, p = 0.041). For those with SPG4 mutations, disease severity was related to the patients’ current age, and the progression rate of disease was positively correlated with age at onset.

**Conclusions:**

SPG4 accounts for most of the AD-HSP cases in Taiwanese, with a frequency significantly higher than in other populations. *SPAST* mutations which predict complete loss of the spastin AAA cassette were associated with an earlier onset of disease.

**Electronic supplementary material:**

The online version of this article (doi:10.1186/s12883-014-0216-x) contains supplementary material, which is available to authorized users.

## Background

Hereditary spastic paraplegias (HSPs) are a heterogeneous group of neurodegenerative disorders clinically characterized by progressive spasticity and weakness of the lower limbs [1]. They can be classified according to the mode of inheritance, or by the absence (“pure form”) or presence (“complicated form”) of additional neurological or systemic manifestations such as dementia, epilepsy, mental retardation, thin corpus callosum, cerebellar ataxia, peripheral neuropathy, deafness, retinopathy, and optic atrophy [1,2]. Pure forms of HSPs are usually inherited as an autosomal dominant trait, whereas most complicated HSPs are autosomal recessive forms. To date, more than 70 loci for HSPs have been mapped and at least 55 genes have been identified [3].

Autosomal dominant HSPs (AD-HSPs) represent around 70% of cases of uncomplicated HSPs [4]. SPG4 is the most common form of AD-HSP, accounting for around 40% of the families in previous reports [2,5]. Spastin, the protein encoded by the SPG4 gene, is a member of the family of AAA proteins (*A*TPase *a*ssociated with diverse cellular *a*ctivities) which share a common functional domain called the AAA cassette [6]. SPG3A is the second most frequent form of AD-HSP, representing approximately 7-10% of all AD-HSP families [7,8]. The SPG3A gene encodes the protein atlastin-1 which is structurally homologous to members in the dynamin superfamily of GTPases [9]. SPG31 has been suggested to be the third most common cause of AD-HSP, with an overall frequency of 2.3-6.5% in the kindreds of primarily European descent [10,11]. The SPG31 gene encodes receptor expression-enhancing protein 1 which may be involved in a chaperone-like function [10].

We previously reported four Taiwanese families with SPG4-related HSP [12]. In this study, we extended the scope of the investigation to screen SPG4, SPG3A and SPG31 in additional AD-HSP families. The results showed an unexpectedly high frequency of SPG4 in the cohort of Taiwanese AD-HSP families.

## Methods

The study protocol was approved by the Chang Gung Memorial Hospital Institutional Review Board (No. 100-4460C). Clinical and genetic features of the participants were examined after written informed consent had been obtained from participants or a parent while participants were children.

### Patients and clinical examinations

This study included 20 unrelated AD-HSP kindreds (49 cases in total) of Han Taiwanese ethnicity collected from the Departments of Neurology, Rehabilitation and Pediatric, Chang Gung Memorial Hospitals, Taiwan (Additional file 1: Figure S1). Families 1 to 4 have been reported previously [12]. A diagnosis of AD-HSP was based on the following diagnostic criteria: (1) spastic paraplegia, or (2) spastic tetraparesis with earlier and more severe affliction of the lower limbs, or (3) spastic paraplegia as an early and prominent sign of a degenerative disease affecting the nervous system; (4) positive family history of spastic paraplegia with affected members in at least two generations; and (5) no other causes of the presenting symptoms [4]. Family history was recorded according to the patients’ reports, and inheritance was ascertained in 14 families by examining affected family members. Clinical information was collected and neurological examinations were performed. Ambulatory disability was scored based on a five-point scale: 0 asymptomatic, 1 minimally impaired (able to run), 2 mildly impaired (able to walk independently but not run), 3 moderately impaired (walk with an aid), and 4 severely impaired (wheelchair bound).

### Genetic studies

Genomic DNA was extracted from peripheral blood leukocytes for genetic analysis, and mutation screenings were conducted in the following order. First, nucleotide substitutions and small deletions or insertions in all exons and their adjacent splicing sites of the SPG4 gene (*SPAST*) were analyzed by polymerase chain reactions and direct sequencing. If a mutation was not detected, multiplex ligation-dependent probe amplification (MLPA) was performed to detect exonic deletions in *SPAST*. Finally, for the cases with no detected mutations in *SPAST*, sequence analysis and MLPA of the SPG3A gene (*ATL1*) and sequence analysis of the SPG31 gene (*REEP1*) were performed. Detected mutations were checked in 100 control DNA samples collected from unrelated ethnic Han Taiwanese.

MLPA was performed using SALSA MLPA kits (P165-B1 HSP probemix; MRC-Holland, Amsterdam, Netherlands) according to the manufacturer’s instructions. For each sample, a normalized value ratio for a relative peak area between 0.8 and 1.2 was considered normal. A heterozygous deletion was expected with a ratio between 0.3 and 0.7, and a heterozygous duplication between 1.3 and 1.7. Total mRNA from the blood leukocytes was extracted using Trizol procedure (Invitrogen, Carlsbad, CA, United States). mRNA analysis was performed as described previously [12] to assess alternations in gene transcription due to *SPAST* mutations.

### Bioinformatic analysis

Novelty of a detected mutation was checked with the Human Gene Mutation Database [13]. Amino acid conservation in orthologues was assessed using the HomoloGene database [14]. Prediction of pathogenicity of a missense mutation was analysed using the PolyPhen-2 program [15].

### Statistical analysis

Data were presented as mean ± SD unless otherwise specified. Disease severity was defined as “mild” with an ambulation score of 1 or 2, or “severe” with a score of 3 or 4. The progression rate of the disease was assessed with a disease-progression score (DPS) calculated as the current disability score divided by disease duration. Mutations were classified according to the consequence, namely, whether they caused a complete loss of the AAA cassette (i.e. nonsense and frameshift mutations proximal to the encoding sequence of the AAA cassette, whole gene deletion, and mRNA decay) or not. Age at onset (AAO) with respect to mutation consequence was tested with t-tests (asymptomatic cases were excluded from the analysis). The relationships between disease severity and current age, gender, disease duration and mutation consequence were tested using the chi-square test or *t*-test. The relationships between DPS and AAO, gender and mutation consequence were tested using the Pearson test or *t*-test. Independence of the association between disease severity (or DPS) and clinical and genetic factors was examined using logistic (or linear) regression analysis. A p value <0.05 was considered to be statistically significant.

## Results

### Mutation detection

**Figure 1:**
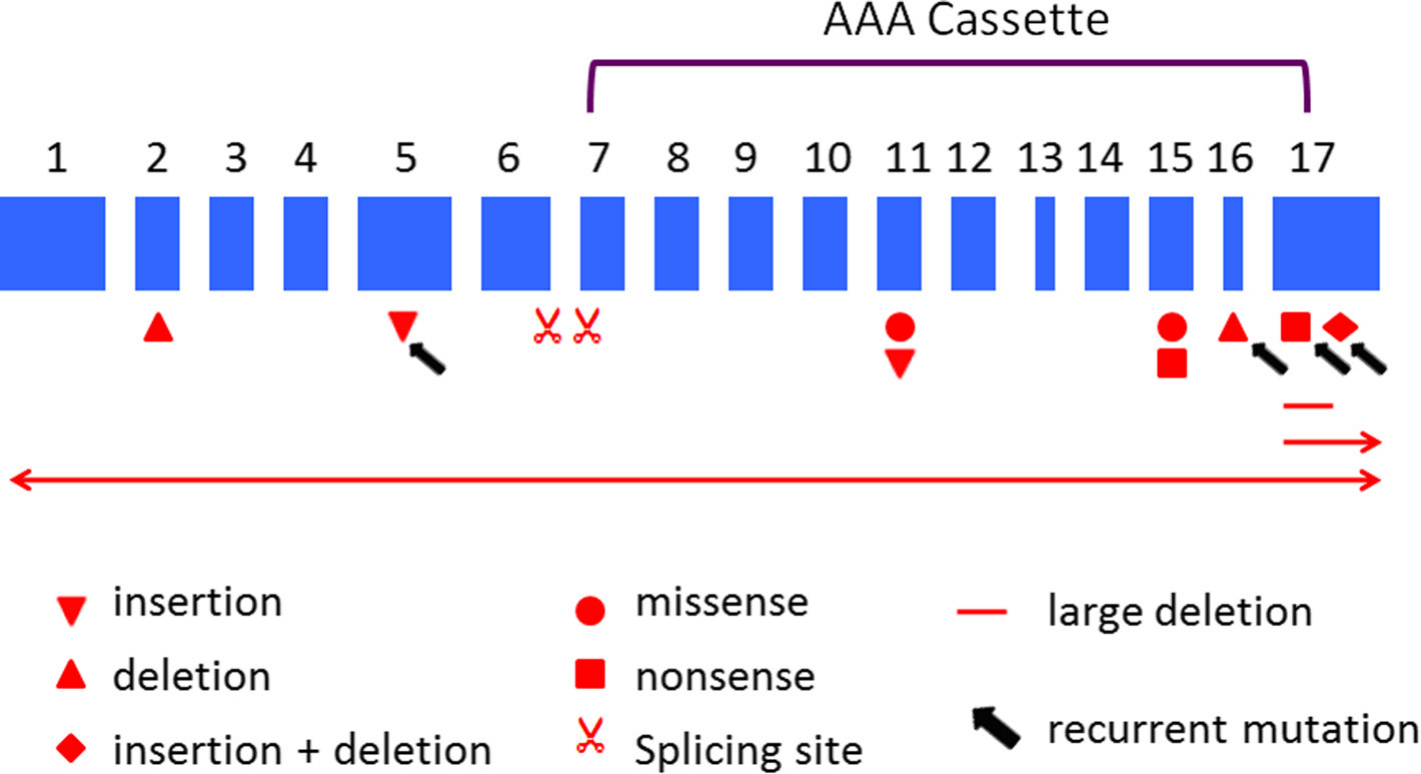
**SPAST mutations detected in the current study.** Schematic representation of the location of *SPAST* mutations detected in this study. Numbered boxes indicate transcript coding regions in exons drawn to scale. Encoding region of the AAA cassette is highlighted.

A total of 14 different *SPAST* mutations were identified in 18 families (Table 1). In this cohort, SPG4 accounted for 90% of the AD-HSP families. Mutation types were quite heterogeneous, including nonsense (2), missense (2), insertion (2), deletion (2), insertion-deletion (1), splicing site (2), and large exonic deletion (3) (Figure 1). Four mutations (c.1361_1363 ins GGG, c.1005-1 G > C, c.1004 + 1 G > T, and c.1738_1740 del ATT/ins GA) were novel mutations. These mutations were neither included in the data derived from the 1000 Genome Project (http://www.1000genomes.org/) [16] nor detected in the 100 control DNA samples. The residue leucine affected by the missense mutation p.L461P and the residue aspartate affected by D555G were highly evolutionarily conserved according to the public genome database (Additional file 2: Figure S2). The substitutions of amino acids caused by the two missense mutations were predicted to be damaging (a score of 1.000 and 0.988 respectively) by the PolyPhen-2 program. In general, nonsense and missense mutations were located in the 3′ half of *SPAST* exons, while frameshift (mini-insertions and mini-deletions) and splicing site mutations were more evenly distributed in the gene. Co-segregation of mutations with the disease was confirmed in 14 families, in which at least two affected members underwent genetic testing. Mutations in *SPAST*, *ATL1* and *REEP1* were not detected in two families (Families 14 and 19).

**Table 1:** **Summary of the families in the current study**

**Family**	**Sex**	**Age (decade)**	**Age at onset (decade)**	**Ambulatory function** ^**†**^	**DPS**	***SPAST*** **mutation**	**Mutation location**	**Presumed effect of the mutation**
1^‡^	F	6	2	2	0.50	c.1714_1715 del AT (p.M572VfsX3)^§^	Exon 16	Protein truncation
	F*	4	2	2	1.00			
2^‡^	F*	7	5	3	1.58	Deletion of 5′- region of exon 17 (p.M577DfsX16)	Exon 17	Protein truncation
	F	4	2	2	0.74			
	M	1	1	1	2.00			
3^‡^	M*	7	5	4	1.82	c.1382 T > C (p.L461P)	Exon 11	Missense
	M	6	6	2	4.00			
	M	6	5	3	3.00			
	M	4	-	0	0.00			
	F	3	3	1	3.33			
	M	3	2	3	5.00			
	F	3	-	0	0.00			
4^‡^	F	8	6	4	1.90	Deletion of exon 17	Exon 17	Protein truncation?
	M*	6	5	3	3.75			
	F	5	5	2	5.00			
5	M*	7	2	3	0.70	Deletion of the whole gene	Exon 1-17	No mRNA production
6	M	5	-	0	0	c.1361_1363 ins GGG^✰^	Exon 11	mRNA decay
	M*	2	1	1	1.00			
	M	1	1	1	1.67			
7	F*	6	5	2	1.82	c.1714_1715 del AT (p.M572VfsX3)^§^	Exon 16	Protein truncation
	M	4	1	2	0.67			
	F	5	1	1	0.29			
	M	2	1	1	0.83			
	M	5	2	2	0.57			
8	F*	6	5	3	3.00	c.1684 C > T (p.R562X)	Exon 15	Protein truncation
9	M	4	3	3	2.31	c.1005-1 G > C (p.N335NfsX2)^✰^	Intron 6	Protein truncation
	F	1	1	1	2.00			
	M*	1	1	1	2.00			
10	F	8	4	1	0.28	c.1004 + 1 G > T (p.G291WfsX4)^✰^	Intron 6	Protein truncation
	M	6	1	1	0.24			
	F*	5	2	1	0.34			
	M	1	1	2	2.86			
11	F*	5	4	2	2.00	c.1738_1740 del ATT/ins GA (p.I580DfsX9)^¶✰^	Exon 17	Protein truncation
12	M*	6	4	3	1.50	c.1664 A > G (p.D555G)	Exon 15	Missense
13	F	7	6	3	2.00	c.448_451 del AAGA (p.K150KfsX9)	Exon 2	Protein truncation
	M*	5	1	3	0.77			
14	M*	6	5	2	5.00	Not detected		Not applicable
15	F	8	7	3	6.00	c.1741 C > T (p.R581X)^∥^	Exon 17	Protein truncation
	M*	6	5	2	1.54			
	M	5	5	1	2.00			
16	F	7	5	3	1.58	c.730 ins T, c.732_733 ins CA (p.N244X)^#^	Exon 5	Protein truncation
	M*	6	5	3	2.50			
17	F	7	7	2	4.00	c.1741 C > T (p.R581X)^∥^	Exon 17	Protein truncation
	M*	5	3	3	2.31			
18	M*	7	3	2	0.54	c.730 ins T, c.732_733 ins CA (p.N244X)^#^	Exon 5	Protein truncation
	M	6	4	3	1.88			
19	M*	3	2	1	1.67	Not detected		Not applicable
20	F	6	5	3	3.33	c.1738_1740 del ATT/ins GA (p.I580DfsX9)^¶✰^	Exon 17	Protein truncation
	M	6	4	2	1.54			

*Abbreviations*: *DPS* disease progression score, *F* female, *M* male.

*proband.

^†^0, asymptomatic; 1, able to run; 2, unable to run, walking independently; 3, walking with an aid; 4, wheelchair bound.

^‡^reported previously [12].

^§¶∥#^identical mutations.

^✰^novel mutations.

### *SPAST* mRNA analysis

For the in-frame insertion c.1361_1363 ins GGG detected in Family 6, only the wild type of mRNA was detected in mRNA analysis and the decay of mutant mRNA is suspected (Additional file 3: Figure S3). For the other insertions and mutations of missense, nonsense, and deletion types, mRNA analysis showed nucleotide changes identical to those in genomic DNA. The two splicing site mutations in intron 6 led to skipping of transcription of the first 8 bases of exon 7 (Family 9) and the whole of exon 6 (Family 10), respectively. For the exonic deletion revealed by MLPA in Family 2, cDNA analysis showed a deletion of 1090 bases at the 5′-end of exon 17, as reported previously [12]. In Family 4, the effects of complete loss of exon 17 on translation could not be determined due to failure to amplify the mutant transcript.

### Clinical presentation

In this AD-HSP cohort, AAO in the probands ranged from 1 to 48 years, and six (30%) families had at least one case whose AAO was less than 10 years. The clinical characteristics of the SPG4 cases are summarized in Table 2. The onset of disease ranged from 1 to 69 years of age. Among the 47 cases carrying *SPAST* mutations, 44 (94%) showed spastic paraparesis and 3 (6%) were asymptomatic. All of the symptomatic SPG4 patients manifested a pure phenotype, except for a case in Family 7 who presented with distal amyotrophy in the upper limbs. The most common clinical presentation was gait disorder due to spasticity or increased tendon reflexes in the lower limbs. Neurological examinations of the cranial nerves and upper limbs were normal except for an accentuated jaw-jerk and tendon reflex in some patients. Considerable variations in AAO, rate of progression or severity of disease were noted, even in mutation carriers from the same family (Table 1). There was no clear phenotype correlation with respect to the different types of mutations. For the two families without detected mutations, one had a pure phenotype while the other presented with deafness in the affected members.

**Table 2:** **Clinical characteristics of the SPG4 patients (n = 47)**

**Men/Women**	**27/20**
Mean age (range), years	44 ± 19 (6 – 74)
Mean age at onset (range), years*	28 ± 19 (1 – 69)
Mean disease duration (range), years*	17 ± 12 (3 – 43)
Clinical features	
Asymptomatic individuals	3/47 (6%)
Increased tendon reflexes in the upper limbs	40/47 (85%)
Increased tendon reflexes in the lower limbs	47/47 (100%)
Presence of extensor plantar reflex	29/45 (64%)
Presence of ankle clonus	23/42 (55%)
Attenuated vibration perception at the ankles	23/45 (51%)
Dysfunction of bladder control^†^	21/46 (46%)
Ambulation function, 0/1/2/3/4	3/12/14/16/2
case No. (%)^‡^	(6%/26%/30%/34%/4%)

*asymptomatic cases not included.

^†^urinary retention, frequency or incontinence.

^‡^0, asymptomatic; 1, able to run; 2, unable to run, walking independently; 3, walking with an aid; 4, wheelchair bound.

### Phenotype determinants

For the SPG4 cases, mutations causing complete loss of the AAA cassette were associated with an earlier onset of disease (20 ± 18 years, n =16) compared with those with preservation of partial or total AAA cassette (32 ± 19 years, n = 28; p = 0.041). In univariate analysis, disease severity was related with the patients’ current age (37 ± 19 years for mild disease vs. 55 ± 12 years for severe disease, p <0.001), and DPS was correlated with AAO (r = 0.564, p <0.001) (Additional file 4: Table S1 and Additional file 5: Table S2). After adjusting for gender, disease duration and consequence of mutation, the association of age (odds ratio 1.10, 95% confidence interval 1.04-1.16 for every increase of 1 year, p = 0.002) with disease severity, and that of AAO (β = 0.038, standard error 0.010, p <0.001) with DPS remained significant. Consequence of *SPAST* mutation was not related with either disease severity or DPS.

## Discussion

Compared to previous series in other populations, the current findings showed a significantly higher frequency of SPG4 for AD-HSP in our ethnically Han Taiwanese cohort (90% vs. 30-60%; Additional file 6: Table S3). Previously, a study of HSP cohort in Sardinia, Italy showed that SPG4 was responsible for all of nine AD-HSP families receiving genetic test [17]. However, eight of these SPG4 families were attributable to a same multi-exonic deletion in *SPAST*, suggesting a founder effect for this mutation in this population. In contrast, the predominance of SPG4 in our cohort could not be due to specific founder mutations because most of the detected mutations were unique to the respective families. Although 7-10% of AD-HSPs are known to be caused by SPG3A, none of our cases had *ATL1* mutations. SPG3A is characterized by early onset of disease, usually before 10 years of age [8]. Failure to detect SPG3A in this study due to ascertainment bias is unlikely in view of the wide range of AAO (1–48 years) in the probands and the inclusion of pediatric cases in this cohort. A possible explanation for this phenomenon may be ethnic differences in the genetic background. However, because of the relatively small kindred number in the present study, this finding needs to be confirmed with a larger cohort of AD-HSP families.

The current study showed that missense mutations were located in the AAA cassette-encoding region, while frameshift mutations which create premature termination codons were scattered along the *SPAST* gene (Figure 1). In spite of the heterogeneity in mutation types, all of these mutations may either alter the structure or disrupt the integrity of the AAA cassette. Further, most of these mutations were predicted to produce a truncated spastin or absence of mRNA transcription. This finding suggests that haploinsufficiency due to the abolishment of ATPase activity is the most important pathogenicity for disease-causing *SPAST* mutations.

For SPG4, mutations causing a complete loss of the spastin AAA cassette were associated with earlier onset of disease than those with at least partial AAA cassette preservation. However, further studies of the production of truncated spastin proteins by the mutations predicting partial AAA loss and function of spastin protein carrying partial AAA cassette in cellular levels will be needed to provide biological evidence for this finding. Except for the common motor dysfunction due to spastic paraparesis, remarkable variations in onset age, progression rate and disease severity were noted in the SPG4 patients, even for those from the same family. We also found that later onset of the disease was associated with faster disease progression. This finding is consistent with Harding’s observation on the pure form AD-HSP [18] and has also been noted in previous studies in SPG4 [19,20]. In addition, the patient’s current age was the most significant determinant for disease severity. Taken together, these findings suggest that the SPG4 phenotype is the product of interactions between *SPAST* mutations and environmental or other genetic factors.

There are some limitations to this study. First, this study was based on a relatively small cohort of AD-HSP kindreds collected from two medical centers in Taiwan. Our findings need to be confirmed by a more extensive nationwide survey of AD-HSP families. Second, this is a cross-sectional study, and disease progression would be more accurately evaluated by a longitudinal observation. Third, the five-graded ambulation score did not have enough sensitivity in detecting minor differences in disease severity. A more detailed assessment scale, such as Spastic Paraplegia Rating Scale [21], would be better in reflecting overall functional impairment. Fourth, because the age of symptom onset was determined according to patient reports, there could be errors in recalling the information, especially for those with long-lasting disease. Finally, this study did not test the genetic modifiers, such as some sequence variants in *ATL1* and *HSPD1*, which have been postulated to account for the variability in SPG4 phenotypes [22].

## Conclusions

SPG4 accounted for a significantly higher proportion of AD-HSPs in ethnic Taiwanese than in other populations. There was no clear genotype-phenotype correlation among the SPG4 patients, except that mutations resulting in complete loss of the spastin AAA cassette were associated with an earlier onset of disease.

## Additional files

Additional file 1: Figure S1. Pedigrees of the 20 HSP families in the study. The pedigrees of the 20 hereditary spastic paraplegia families included in the study. The circles represent female subjects and the squares represent male subjects, and the diamond represents the patient whose gender was withheld for confidentiality reasons. The filled symbols indicate affected individuals and the question marks indicate uncertain disease affection. The cases that were involved in the clinical and genetic studies are indicated with a cross bar above the individual symbol. The probands are indicated by arrows. Additional file 2: Figure S2. Conservation of the mutated residues in different species. Conservation of the residues affected by the mutations p.L461P and p.D555G in different species. Additional file 3: Figure S3. cDNA analysis of the mutations. cDNA analysis of the *SPAST* mutations in Family 2 (large deletion of 5′ region of exon 17), Family 9 (spicing acceptor mutation, the boxed sequence representing the nucleotides spliced during transcription due to the mutation) and Family 10 (splicing donor mutation). Additional file 4: Table S1. Determinants of SPG4 disease severity. Univariate and multivariate regression analyses for determinants of disease severity in the SPG4 cases. Additional file 5: Table S2. Determinants of SPG4 disease progression. Univariate and multivariate analyses for determinants of disease progression score in the SPG4 cases. Additional file 6: Table S3. Proportions of SPG4 in different populations. Proportions of SPG4 in autosomal dominant hereditary spastic paraplegias (AD-HSPs) in different populations.

## Abbreviations

AAA: ATPase associated with diverse cellular activities
AAO: Age at onset
AD: Autosomal dominant
DPS: Disease progression score
HSP: Hereditary spastic paraplegia
MLPA: Multiplex ligation-dependent probe amplification
