# Supplementary figures and images for: High frequency of SPG4 in Taiwanese families with autosomal dominant hereditary spastic paraplegia
Source: BMC Neurol. 2014 Nov 25;14:216. doi: 10.1186/s12883-014-0216-x (PMC4254010; doi:10.1186/s12883-014-0216-x)

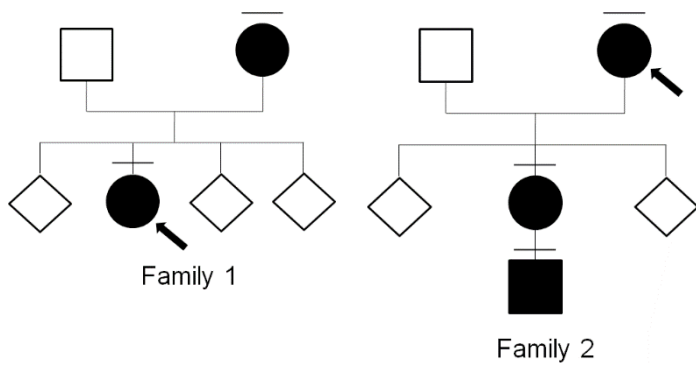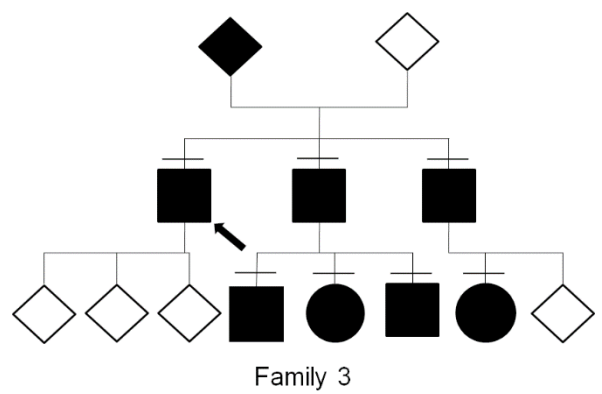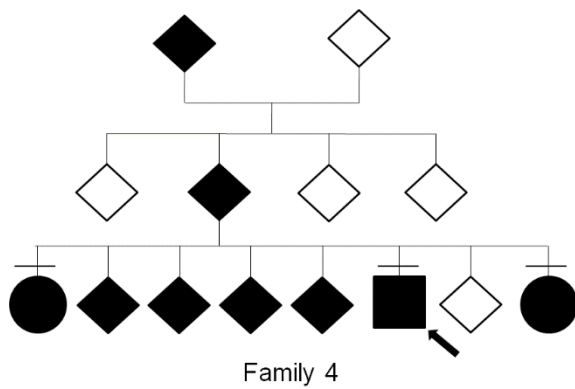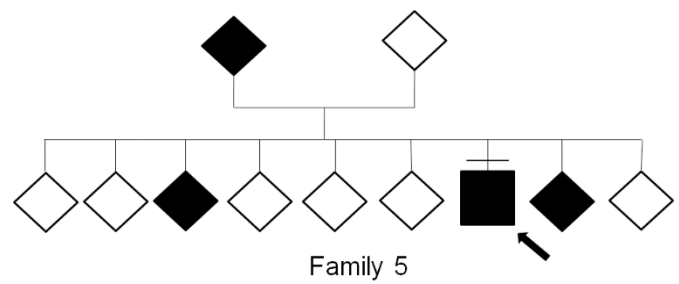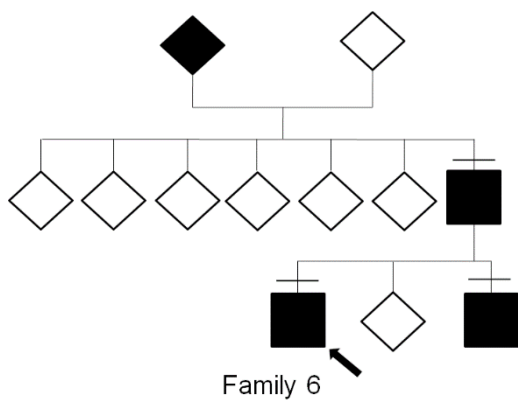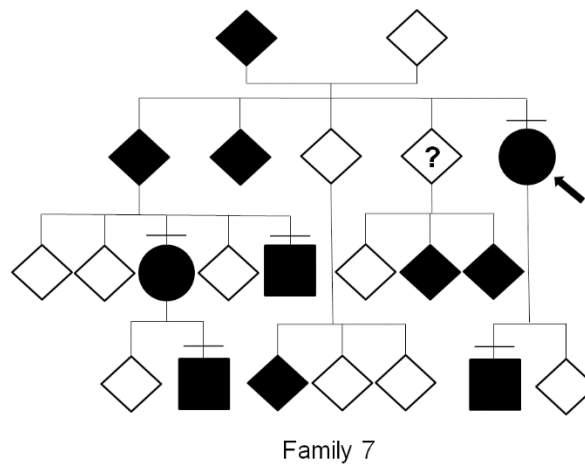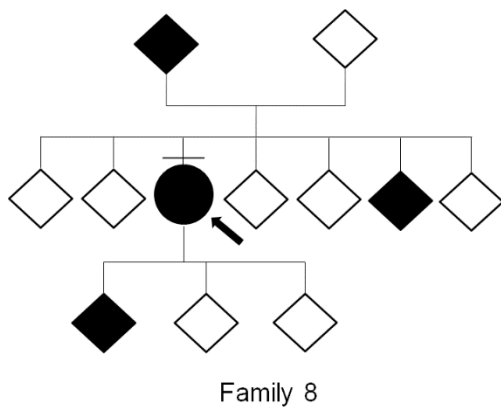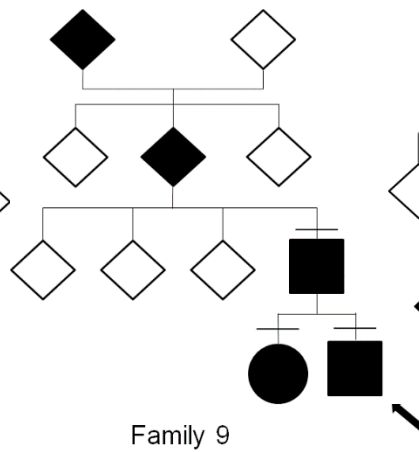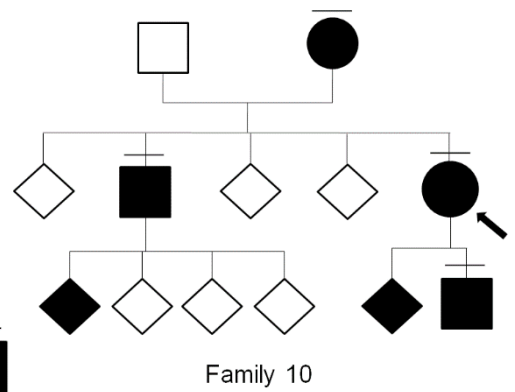

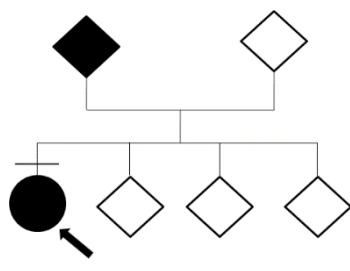

Family 11

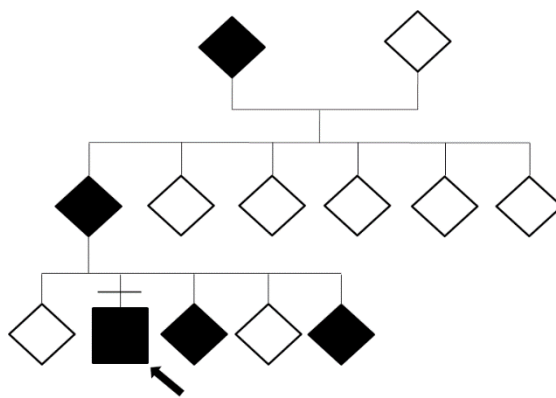

Family 12

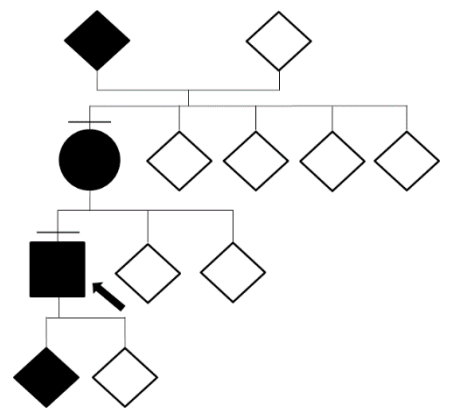

Family 13

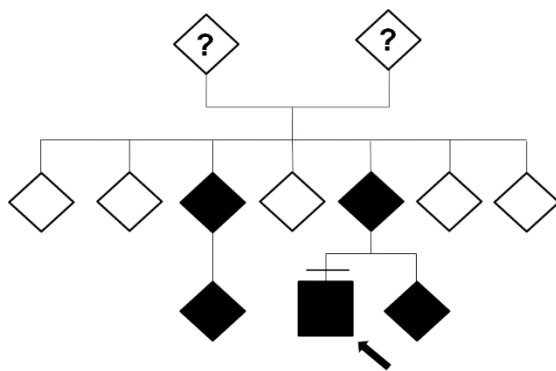

Family 14

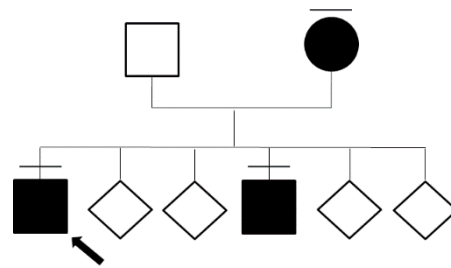

Family 15

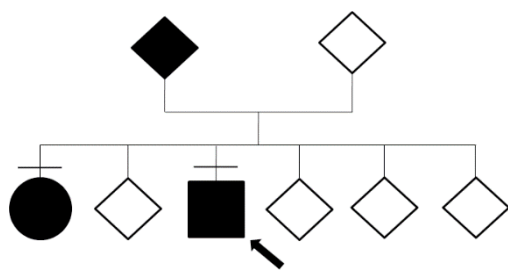

Family 16

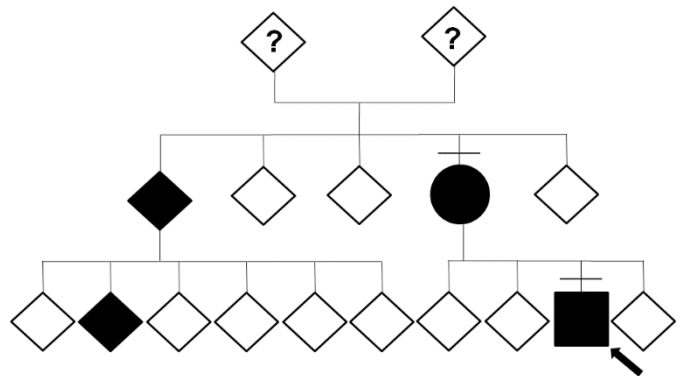

Family 17

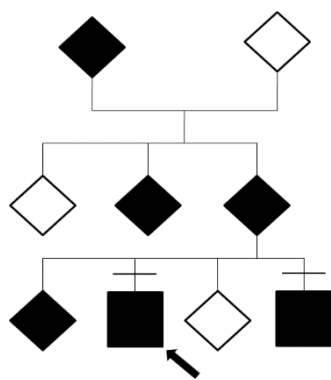

Family 18

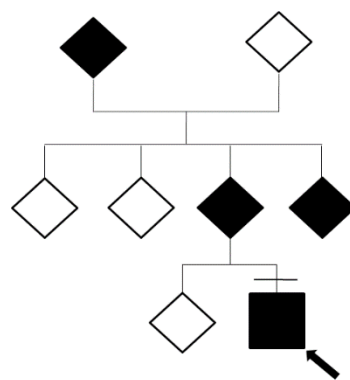

Family 19

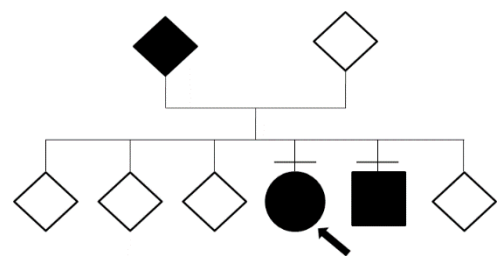

Family 20

Supplement: Additional file 1: Figure S1. — Pedigrees of the 20 HSP families in the study. The pedigrees of the 20 hereditary spastic paraplegia families included in the study. The circles represent female subjects and the squares represent male subjects, and the diamond represents the patient whose gender was withheld for confidentiality reasons. The filled symbols indicate affected individuals and the question marks indicate uncertain disease affection. The cases that were involved in the clinical and genetic studies are indicated with a cross bar above the individual symbol. The probands are indicated by arrows. [file 12883_2014_216_MOESM1_ESM.pdf]

## Family 2

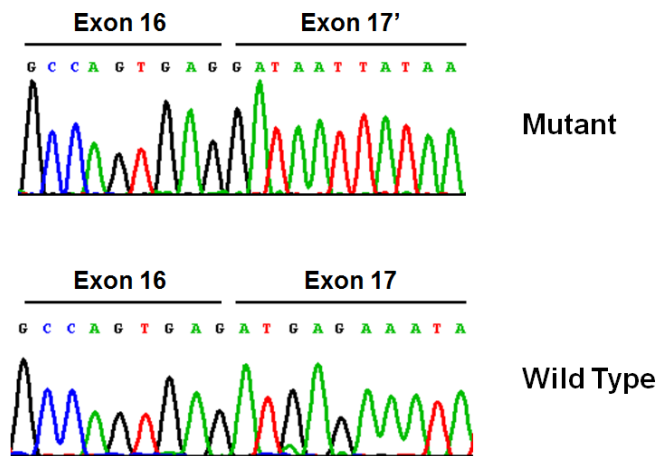

## Family 9

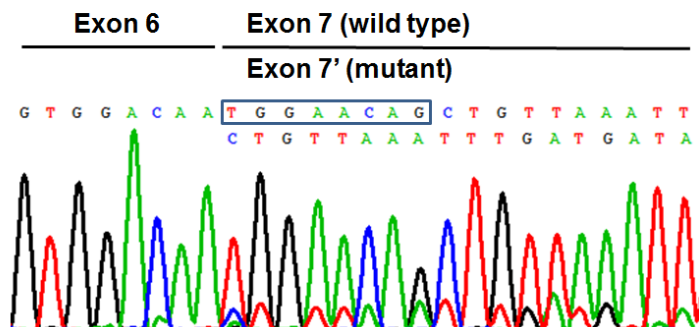

## Family 10

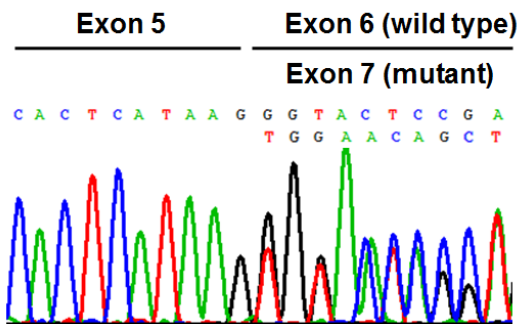

Supplement: Additional file 3: Figure S3. — cDNA analysis of the mutations. cDNA analysis of the SPAST mutations in Family 2 (large deletion of 5′ region of exon 17), Family 9 (spicing acceptor mutation, the boxed sequence representing the nucleotides spliced during transcription due to the mutation) and Family 10 (splicing donor mutation). [file 12883_2014_216_MOESM3_ESM.pdf]
