# Supplementary material for: High frequency of SPG4 in Taiwanese families with autosomal dominant hereditary spastic paraplegia
Source: BMC Neurol. 2014 Nov 25;14:216. doi: 10.1186/s12883-014-0216-x (PMC4254010; doi:10.1186/s12883-014-0216-x)
Supplement: Additional file 2: Figure S2. — Conservation of the mutated residues in different species. Conservation of the residues affected by the mutations p.L461P and p.D555G in different species. [file 12883_2014_216_MOESM2_ESM.pdf]

Species	p.L461P	p.D555G
Homo sapiens	EGEHDASRR**L**KTEFLIEFD	GSDLTALAK**D**AALGPIREL
Pan troglotytes	EGEHDASRR**L**KTEFLIEFD	GSDLTALAK**D**AALGPIREL
Bos Taurus	EGEHDASRR**L**KTEFLIEFD	GSDLTALAK**D**AALGPIREL
Canis lupus	EGEHDASRR**L**KTEFLIEFD	GSDLTALAK**D**AALGPIREL
Rattus norvegicus	EGEHDASRR**L**KTEFLIEFD	GSDLTALAK**D**AALGPIREL
Mus musculus	EGEHDASRR**L**KTEFLIEFD	GSDLTALAK**D**AALGPIREL
Gallus gallus	EGEHDASRR**L**KTEFLIEFD	GSDLTALVK**D**AALGPIREL
Danio rerio	EGEHDASRR**L**KTEFLIEFD	GSDLTSLAK**D**AALGPIREL
