# Supplementary material for: High frequency of SPG4 in Taiwanese families with autosomal dominant hereditary spastic paraplegia
Source: BMC Neurol. 2014 Nov 25;14:216. doi: 10.1186/s12883-014-0216-x (PMC4254010; doi:10.1186/s12883-014-0216-x)
Supplement: Additional file 4: Table S1. — Determinants of SPG4 disease severity. Univariate and multivariate regression analyses for determinants of disease severity in the SPG4 cases. [file 12883_2014_216_MOESM4_ESM.doc]

**Additional file 4: Table S1.** Univariate and multivariate regression analyses for determinants of disease severity in the SPG4 cases (n= 47)

(a) Univariate analysis

| Variable | Disease Severity | | p |
| --- | --- | --- | --- |
|  | Mild | Severe |  |
| Age (years) | 37 ± 19**^*^** | 55 ± 12 | < 0.001 |
| Male gender (%) | 55 | 61 | 0.689 |
| Disease duration (years) | 17 ± 14 | 17 ± 10 | 0.867 |
| Complete AAA**^†^** loss (%) | 34 | 39 | 0.760 |

(b) Multivariate analysis

| Variable | Odds ratio (95% CI) | p |
| --- | --- | --- |
| Age (year) | 1.10 (1.04-1.16) | 0.002 |
| Male gender | 3.61 (0.69-18.9) | 0.127 |
| Disease duration (year) | 0.94 (0.98-1.15) | 0.121 |
| Complete AAA† loss | 2.67 (0.42-16.7) | 0.295 |

* continuous variables expressed as mean ± standard deviation

† *A*TPase *a*ssociated with diverse cellular *a*ctivities
