# Supplementary material for: High frequency of SPG4 in Taiwanese families with autosomal dominant hereditary spastic paraplegia
Source: BMC Neurol. 2014 Nov 25;14:216. doi: 10.1186/s12883-014-0216-x (PMC4254010; doi:10.1186/s12883-014-0216-x)
Supplement: Additional file 5: Table S2. — Determinants of SPG4 disease progression. Univariate and multivariate analyses for determinants of disease progression score in the SPG4 cases. [file 12883_2014_216_MOESM5_ESM.doc]

**Additional file 5: Table S2.** Univariate and multivariate analyses for determinants of disease progression score in the SPG4 cases (n= 47)

(a) Univariate analysis

| Variable | Disease Progression Score | p |
| --- | --- | --- |
| Age at onset | r**^*^**= 0.564 | < 0.001 |
| Gender |  |  |
| Male | 1.74 ± 1.24**^†^** | 0.487 |
| Female | 2.03 ± 1.64 |
| AAA**^‡^** cassette |  |  |
| Complete loss | 1.33 ± 0.90 | 0.051 |
| Partial or complete preservation | 2.17 ± 1.57 |

(b) Multivariate analysis

| Variable | β estimate (S.E.) | p |
| --- | --- | --- |
| Age at onset (year) | 0.038 (0.010) | < 0.001 |
| Male gender | 0.249 (0.367) | 0.501 |
| Complete AAA**^‡^** loss | -0.483 (0.381) | 0.212 |
| Constant | 0.963 (0.460) | 0.043 |

* correlation coefficient

† continuous variables expressed as mean ± standard deviation

‡ *A*TPase *a*ssociated with diverse cellular *a*ctivities
