# Supplementary material for: High frequency of SPG4 in Taiwanese families with autosomal dominant hereditary spastic paraplegia
Source: BMC Neurol. 2014 Nov 25;14:216. doi: 10.1186/s12883-014-0216-x (PMC4254010; doi:10.1186/s12883-014-0216-x)
Supplement: Additional file 6: Table S3. — Proportions of SPG4 in different populations. Proportions of SPG4 in autosomal dominant hereditary spastic paraplegias (AD-HSPs) in different populations. [file 12883_2014_216_MOESM6_ESM.doc]

**Additional file 6: Table S3.** Proportions of SPG4 in autosomal dominant hereditary spastic paraplegias (AD-HSPs) in different populations

| **Country / Region** | **Number of AD-HSP Families Screened** | **Proportion of SPG4** | **Inclusion of MLPA^*^** | **Supplementary Reference** |
| --- | --- | --- | --- | --- |
| Australia | 24 | 58% | Yes | 1 |
| Brazil | 34 | 35% | Yes | 2 |
| China | 22 | 18% | No | 3 |
| China | 11 | 45% | Yes | 4 |
| Denmark | 42 | 24% | Yes | 5 |
| Europe | 119 | 37% | No | 6 |
| Germany | 63 | 38% | No | 7 |
| Italy | 18 | 44% | No | 8 |
| Italy | 24 | 46% | Yes | 9 |
| Italy | 9 | 100% | Yes | 10 |
| Japan | 12 | 42% | No | 11 |
| Japan | 8 | 38% | No | 12 |
| Korea | 11 | 64% | No | 13 |
| Korea | 15 | 67% | Yes | 14 |
| North America | 49 | 31% | No | 15 |
| Norway | 35 | 46% | Yes | 16 |
| Portugal | 61 | 20% | No | 17 |
| Portugal | 89 | 34% | No | 18 |
| Romania | 23 | 70% | No | 19 |
| Spain | 141 | 31% | Yes | 20 |
| Taiwan**^†^** | 20 | 90% | Yes | - |

*multiplex ligation-dependent probe amplification

† the current study

**Supplementary References**

1. Vandebona H, Kerr NP, Liang C, Sue CM. SPAST mutations in Australian patients with hereditary spastic paraplegia. Intern Med J 2012;**42**:1342-7.

2. França M Jr, Dogini D, D'Abreu A, Teive H, Munhoz R, Raskin S, Moro A, Melo C, Gomes A, Saute J, Jardim L, Lopes-Cendes I. SPG4-related hereditary spastic paraplegia: frequency and mutation spectrum in Brazil. Clin Genet 2014;**86**:194-6.

3. Tang B, Zhao G, Xia K, Pan Q, Luo W, Shen L, Long Z, Dai H, Zi X, Jiang H. Three novel mutations of the spastin gene in Chinese patients with hereditary spastic paraplegia. Arch Neurol 2004;**61**:49-55.

4. Fei QZ, Tang WG, Rong TY, Tang HD, Liu JR, Guo ZL, Fu Y, Xiao Q, Wang XJ, He SB, Cao L, Chen SD. Two novel mutations in the Spastin gene of Chinese patients with hereditary spastic paraplegia. Eur J Neurol 2011;**18**:1194-6.

5. Svenstrup K, Bross P, Koefoed P, Hjermind LE, Eiberg H, Born AP, Vissing J, Gyllenborg J, Nørremølle A, Hasholt L, Nielsen JE. Sequence variants in SPAST, SPG3A and HSPD1 in hereditary spastic paraplegia. J Neurol Sci 2009;**284**:90-5.

6. Fonknechten N, Mavel D, Byrne P, Davoine CS, Cruaud C, Bönsch D, Samson D, Coutinho P, Hutchinson M, McMonagle P, Burgunder JM, Tartaglione A, Heinzlef O, Feki I, Deufel T, Parfrey N, Brice A, Fontaine B, Prud'homme JF, Weissenbach J, Dürr A, Hazan J. Spectrum of SPG4 mutations in autosomal dominant spastic paraplegia. Hum Mol Genet 2000;**9**:637-44.

7. Sauter S, Miterski B, Klimpe S, Bönsch D, Schöls L, Visbeck A, Papke T, Hopf HC, Engel W, Deufel T, Epplen JT, Neesen J. Mutation analysis of the spastin gene (SPG4) in patients in Germany with autosomal dominant hereditary spastic paraplegia. Hum Mutat 2002;**20**:127-32.

8. Magariello A, Muglia M, Patitucci A, Mazzei R, Conforti FL, Gabriele AL, Sprovieri T, Ungaro C, Gambardella A, Mancuso M, Siciliano G, Branca D, Aguglia U, de Angelis MV, Longo K, Quattrone A. Novel spastin (SPG4) mutations in Italian patients with hereditary spastic paraplegia. Neuromuscul Disord 2006;**16**:387-90.

9. Magariello A, Muglia M, Patitucci A, Ungaro C, Mazzei R, Gabriele AL, Sprovieri T, Citrigno L, Conforti FL, Liguori M, Gambardella A, Bono F, Piccoli T, Patti F, Zappia M, Mancuso M, Iemolo F, Quattrone A. Mutation analysis of the SPG4 gene in Italian patients with pure and complicated forms of spastic paraplegia. J Neurol Sci 2010;**288**:96-100.

10. Racis L, Tessa A, Di Fabio R, Storti E, Agnetti V, Casali C, Santorelli FM, Pugliatti M. The high prevalence of hereditary spastic paraplegia in Sardinia, insular Italy. J Neurol 2014;**261**:52-9.

11. Yabe I, Sasaki H, Tashiro K, Matsuura T, Takegami T, Satoh T. Spastin gene mutation in Japanese with hereditary spastic paraplegia. J Med Genet 2002;**39**:e46.

12. Basri R, Yabe I, Soma H, Takei A, Nishimura H, Machino Y, Kokubo Y, Kosugi M, Okada R, Yukitake M, Tachibana H, Kuroda Y, Kuzuhara S, Sasaki H. Four mutations of the spastin gene in Japanese families with spastic paraplegia. J Hum Genet 2006;**51**:711-5.

13. Park SY, Ki CS, Kim HJ, Kim JW, Sung DH, Kim BJ, Lee WY. Mutation analysis of SPG4 and SPG3A genes and its implication in molecular diagnosis of Korean patients with hereditary spastic paraplegia. Arch Neurol 2005;**62**:1118-21.

14. Kim TH, Lee JH, Park YE, Shin JH, Nam TS, Kim HS, Jang HJ, Semenov A, Kim SJ, Kim DS. Mutation analysis of SPAST, ATL1, and REEP1 in Korean Patients with Hereditary Spastic Paraplegia. J Clin Neurol 2014;**10**:257-61.

15. Meijer IA, Hand CK, Cossette P, Figlewicz DA, Rouleau GA. Spectrum of SPG4 mutations in a large collection of North American families with hereditary spastic paraplegia. Arch Neurol 2002;**59**:281-6.

16. Erichsen AK, Inderhaug E, Mattingsdal M, Eiklid K, Tallaksen CM. Seven novel mutations and four exon deletions in a collection of Norwegian patients with SPG4 hereditary spastic paraplegia. Eur J Neurol 2007;**14**:809-14.

17. Loureiro JL, Miller-Fleming L, Thieleke-Matos C, Magalhães P, Cruz VT, Coutinho P, Sequeiros J, Silveira I. Novel SPG3A and SPG4 mutations in dominant spastic paraplegia families. Acta Neurol Scand 2009;**119**:113-8.

18. Loureiro JL, Brandão E, Ruano L, Brandão AF, Lopes AM, Thieleke-Matos C, Miller-Fleming L, Cruz VT, Barbosa M, Silveira I, Stevanin G, Pinto-Basto J, Sequeiros J, Alonso I, Coutinho P. Autosomal dominant spastic paraplegias: a review of 89 families resulting from a portuguese survey. JAMA Neurol 2013;**70**:481-7.

19. Orlacchio A, Patrono C, Borreca A, Babalini C, Bernardi G, Kawarai T. Spastic paraplegia in Romania: high prevalence of SPG4 mutations. J Neurol Neurosurg Psychiatry 2008;79:606-7.

20. Alvarez V, Sánchez-Ferrero E, Beetz C, Díaz M, Alonso B, Corao AI, Gámez J, Esteban J, Gonzalo JF, Pascual-Pascual SI, López de Munain A, Moris G, Ribacoba R, Márquez C, Rosell J, Marín R, García-Barcina MJ, Del Castillo E, Benito C, Coto E; Group for the Study of the Genetics of Spastic Paraplegia. Mutational spectrum of the SPG4 (SPAST) and SPG3A (ATL1) genes in Spanish patients with hereditary spastic paraplegia. BMC Neurol 2010;**10**:89.
